# Transparency of Results Reporting in Cancer Clinical Trials

**DOI:** 10.1001/jamanetworkopen.2023.28117

**Published:** 2023-08-09

**Authors:** Jennifer Kao, Joseph S. Ross, Jennifer E. Miller

**Affiliations:** 1 Anderson School of Management, UCLA Center for Health Policy Research, University of California, Los Angeles; 2 Yale School of Medicine, New Haven, Connecticut

## Abstract

This cross-sectional study investigates rates of results reporting among oncology clinical trials across trial registries, medical journals, and medical conferences.

## Introduction

The COVID-19 pandemic highlighted the importance of timely access to clinical trial results for public health. Despite decades-long efforts to improve results reporting for clinical research, problems persist.^1^ Trial investigators have 3 key platforms to disseminate results: trial registries,^2^ medical journals,^3^ and medical conferences. These platforms vary in their accessibility, scope, and depth. Trials presented as abstracts at conferences are limited in word count length and audience (conference attendees). Additionally, while ClinicalTrials.gov offers publicly accessible trial result summaries, journal publications often require payment for more detailed trial reports. Accordingly, we characterized results reporting across these platforms for trials registered in ClinicalTrials.gov completed between 2008 to 2021 with an oncologic indication, the second leading cause of death in the United States.^4^

## Methods

This cross-sectional study followed the STROBE reporting guideline. Per the Common Rule, the study did not need institutional review board approval or informed consent owing to its use of publicly available data.

We identified oncology clinical trials registered in ClinicalTrials.gov and completed between 2008 and 2021, extracting data on their characteristics, including results reporting dates on ClinicalTrials.gov and indexed publications (Figure). We then determined whether trials reported results at any American Society of Clinical Oncology (ASCO) Annual Meeting from 2008 to 2021. We investigated the proportion of interventional phase 2 and 3 trials with results reporting within 1 and 3 years of trial primary completion dates and analyzed factors associated with reported proportions. We used Stata statistical software version 16.1 (StataCorp). Statistical tests were 2-sided, with a significance threshold set at *P* = .05.

**Figure.**
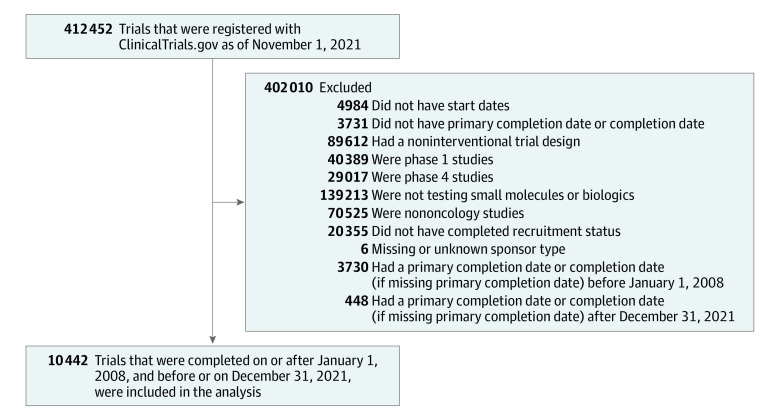
Clinical Trials Included in the Study

## Results

Among 10 442 eligible trials, results reporting was low within 1 year (6.8% in publications, 17.9% on ClinicalTrials.gov, and 18.3% at ASCO meetings) and 3 years (10.5% in publications, 40.0% on ClinicalTrials.gov, and 21.9% at ASCO meetings) of completion (Table). The reporting rates within 1 year were similar for ClinialTrials.gov and ASCO meetings (*P* = .53). However, for reporting at 3 years, ClinicalTrials.gov had a higher rate vs ASCO (*P* < .001). Furthermore, reporting was more common on ClinicalTrials.gov and ASCO vs publications at 1 (*P* < .001) and 3 (*P* < .001) years. Overall, 44 trials (0.4%) reported results across all platforms and 3787 trials (36.3%) reported results on at least 1 platform by 1 year, which increased to 121 trials (1.2%) and 5853 trials (56.1%) at 3 years.

**Table.** Characteristics of Clinical Trials by Dissemination Platform and Reporting Year

Characteristic	All trials, No. (column %) (N = 10 442)^b^	Trials reporting results, No. (row %)^a^									
		In ClinicalTrials.gov		At ASCO		In a publication		In all platforms		In ≥1 platform	
		By 1 y (n = 1873 [17.9%])	By 3 y (n = 4181 [40.0%])	By 1 y (n = 1906 [18.3%])	By 3 y (n = 2291 [21.9%])	By 1 y (n = 710 [6.8%])	By 3 y (n = 1095 [10.5%])	By 1 y (n = 44 [0.4%])	By 3 y (n = 121 [1.2%])	By 1 y (n = 3787 [36.3%])	By 3 y (n = 5853 [56.1%])
Funding source^c^											
Industry	3618 (34.6)	916 (25.3)	1748 (48.3)	874 (24.2)	1090 (30.1)	136 (3.8)	238 (6.6)	23 (0.6)	59 (1.6)	1563 (43.2)	2269 (62.7)
NIH	627 (6.0)	147 (23.4)	413 (65.9)	122 (19.5)	138 (22.0)	53 (8.5)	79 (12.6)	4 (0.6)	12 (1.9)	262 (41.8)	460 (73.4)
Other^d^	6197 (59.3)	8107 (13.1)	2020 (32.6)	910 (14.7)	1063 (17.2)	521 (8.4)	778 (12.6)	17 (0.3)	50 (0.8)	1962 (31.7)	3124 (50.4)
Primary purpose											
Treatment	9465 (90.6)	1731 (18.3)	3813 (40.3)	1858 (19.6)	2229 (23.5)	648 (6.8)	995 (10.5)	42 (0.4)	117 (1.2)	3557 (37.6)	5405 (57.1)
Prevention	352 (3.4)	48 (13.6)	121 (34.4)	11 (3.1)	12 (3.4)	25 (7.1)	40 (11.4)	0	0	74 (21.0)	149 (42.3)
Other^e^	577 (5.5)	88 (15.3)	235 (40.7)	34 (5.9)	45 (7.8)	36 (6.2)	58 (10.1)	2 (0.3)	4 (0.7)	147 (25.5)	284 (49.2)
Unknown	44 (0.4)	6 (13.6)	12 (27.3)	2 (4.5)	4 (9.1)	1 (2.3)	2 (4.5)	0	0	8 (18.2)	14 (31.8)
Intervention group											
Drug	9762 (93.5)	1761 (18.0	3924 (40.2)	1805 (18.5)	2166 (22.2)	659 (6.8)	1020 (10.4)	40 (0.4)	115 (1.2)	3570 (36.6)	5504 (56.4)
Biologic	1462 (14.0)	265 (18.1)	644 (44.0)	279 (19.1)	337 (23.1)	129 (8.8)	192 (13.1)	9 (0.6)	20 (1.4)	558 (38.2)	884 (60.5)
Phase											
1-2	1504 (14.4)	238 (15.8)	564 (37.5)	347 (23.1)	388 (25.8)	112 (7.4)	146 (9.7)	8 (0.5)	22 (1.5)	580 (38.6)	825 (54.9)
2	6288 (60.2)	1143 (18.2)	2578 (41.0)	1139 (18.1)	1342 (21.3)	399 (6.3)	645 (10.3)	24 (0.4)	64 (1.0)	2291 (36.4)	3606 (57.3)
2-3	210 (2.0)	16 (7.6)	44 (21.0)	37 (17.6)	41 (19.5)	19 (9.0)	25 (11.9)	1 (0.5)	1 (0.5)	61 (29.0)	85 (40.5)
3	1817 (17.4)	409 (22.5)	823 (45.3)	370 (20.4)	503 (27.7)	143 (7.9)	228 (12.5)	11 (0.6)	34 (1.9)	751 (41.3)	1126 (62.0)
No. of patients enrolled											
Low (<100)	7096 (68)	1165 (16.4)	2742 (38.6)	1045 (14.7)	1222 (17.2)	458 (6.5)	709 (10.0)	16 (0.2)	47 (0.7)	2336 (32.9)	3783 (53.3)
Medium (100-500)	2625 (25.1)	531 (20.2)	1110 (42.3)	660 (25.1)	797 (30.4)	168 (6.4)	269 (10.2)	22 (0.8)	58 (2.2)	1092 (41.6)	1584 (60.3)
High (>500)	693 (6.6)	177 (16.9)	329 (47.5)	201 (29.0)	272 (39.2)	72 (10.4)	105 (15.2)	6 (0.9)	16 (2.3)	347 (50.1)	474 (68.4)
Unknown	28 (0.3)	0	0	0	0	12 (42.9)	12 (42.9)	0	0	12 (42.9)	12 (42.9)
Location											
US site	5868 (56.2)	1514 (25.8)	3489 (59.5)	1323 (22.5)	1532 (26.1)	444 (7.6)	666 (11.3)	39 (0.7)	108 (1.8)	2670 (45.5)	4231 (72.1)
Non-US site	4382 (42.0)	350 (8.0)	663 (15.1)	579 (13.2)	754 (17.2)	247 (5.6)	405 (9.2)	5 (0.1)	13 (0.3)	1086 (24.8)	1568 (35.8)
Unknown	192 (1.8)	9 (4.7)	29 (15.1)	4 (2.1)	5 (2.6)	19 (9.9)	24 (12.5)	0	0	31 (16.1)	54 (28.1)

Abbreviations: ASCO, American Society of Clinical Oncology; NIH, National Institutes of Health.

^a^ Percentages are out of the total number of trials per characteristic.

^b^ Percentages are out of the total number of trials in the sample (10 442 trials).

^c^ The funding source was derived from data about the study lead sponsor.

^d^ Other funding sources include other government institutions, academic institutions, individual investigators, research networks, ambiguous institutions, and other institutions.

^e^ Other primary purposes include basic science; device feasibility; diagnostic use; educational, counseling, or training use; health services research; screening; supportive care; and other.

Results reporting on at least 1 platform was similar across industry- and National Institutes of Health (NIH)–funded trials at 1 year (43.2% vs 41.8%; *P* = .51). However, results reporting rates among NIH-funded trials were higher at 3 years (62.7% vs 73.4%; *P* < .001).

## Discussion

This cross-sectional study found that one-third of oncology clinical trials reported results in at least 1 of 3 platforms (ClinicalTrials.gov, publications, or ASCO Annual Meetings) within 1 year of completion and just over half within 3 years. NIH-funded trials had higher results-reporting rates compared with trials sponsored by other funders. Results were more likely to be reported on ClinicalTrials.gov compared with in publications or at ASCO meetings. Given the importance of detailed results reporting and peer review facilitated through journal publication, our results suggest that efforts may be needed to understand low rates of publication observed.

Limitations included that we could not rule out that results reporting occurred in other platforms, including preprints, press releases, and clinical study reports released by regulators like Health Canada. Our findings may not generalize to postmarketing studies; previous studies have found higher results reporting in such studies.^5^ Our findings echo previous studies on clinical research reporting, suggesting insufficient progress by investigators and peer-reviewers in addressing key barriers, such as prioritizing reporting of all results, including inconclusive findings.^6^ More efforts are needed to improve access to clinical trial results to advance patient care, innovation, and the protection of individuals involved in clinical research.

## Notes

Supplement.
Data Sharing Statement
