# Supplementary material for: Transparency of Results Reporting in Cancer Clinical Trials
Source: JAMA Netw Open. 2023 Aug 9;6(8):e2328117. doi: 10.1001/jamanetworkopen.2023.28117 (PMC10413165; doi:10.1001/jamanetworkopen.2023.28117)
Supplement: Supplement. — Data Sharing Statement [file jamanetwopen-e2328117-s001.pdf]

Data Sharing Statement

Kao. Transparency of Results Reporting in Cancer Clinical Trials. *JAMA Netw Open*. Published online August 9, 2023. doi:10.1001/jamanetworkopen.2023.28117

Data
Data available: Yes
Data types: Data (not involving human participants)
How to access data: The clinical trial and publication data that support the findings of this study will be made publicly available at https://github.com/JenKao/TrialTransparency . Clinical outcomes data comes from American Society of Clinical Oncology (ASCO) conference abstracts. This data can be purchased from the ASCO Data Library (https://old- prod.asco.org/research-data/asco-data-library).

When available: With publication

Supporting Documents
Document types: None

Additional Information
Who can access the data: public
Types of analyses: any purpose
Mechanisms of data availability: public repository
Any additional restrictions: none
